# Fetal Growth versus Birthweight: The Role of Placenta versus Other Determinants

**DOI:** 10.1371/journal.pone.0039324

**Published:** 2012-06-18

**Authors:** Marie Cecilie Paasche Roland, Camilla M. Friis, Nanna Voldner, Kristin Godang, Jens Bollerslev, Guttorm Haugen, Tore Henriksen

**Affiliations:** 1 Department of Obstetrics, Oslo University Hospital, Oslo, Norway; 2 Department of Specialized Endocrinology, Oslo University Hospital, Oslo, Norway; 3 University of Oslo, Oslo, Norway; University of Southampton, United Kingdom

## Abstract

**Introduction:**

Birthweight is used as an indicator of intrauterine growth, and determinants of birthweight are widely studied. Less is known about determinants of deviating patterns of *growth* in utero. We aimed to study the effects of maternal characteristics on both birthweight and fetal growth in third trimester and introduce placental weight as a possible determinant of both birthweight and fetal growth in third trimester.

**Methods:**

The STORK study is a prospective cohort study including 1031 healthy pregnant women of Scandinavian heritage with singleton pregnancies. Maternal determinants (age, parity, body mass index (BMI), gestational weight gain and fasting plasma glucose) of birthweight and fetal growth estimated by biometric ultrasound measures were explored by linear regression models. Two models were fitted, one with only maternal characteristics and one which included placental weight.

**Results:**

Placental weight was a significant determinant of birthweight. Parity, BMI, weight gain and fasting glucose remained significant when adjusted for placental weight. Introducing placental weight as a covariate reduced the effect estimate of the other variables in the model by 62% for BMI, 40% for weight gain, 33% for glucose and 22% for parity. Determinants of fetal growth were parity, BMI and weight gain, but not fasting glucose. Placental weight was significant as an independent variable. Parity, BMI and weight gain remained significant when adjusted for placental weight. Introducing placental weight reduced the effect of BMI on fetal growth by 23%, weight gain by 14% and parity by 17%.

**Conclusion:**

In conclusion, we find that placental weight is an important determinant of both birthweight and fetal growth. Our findings indicate that placental weight markedly modifies the effect of maternal determinants of both birthweight and fetal growth. The differential effect of third trimester glucose on birthweight and growth parameters illustrates that birthweight and fetal growth are not identical entities.

## Introduction

Birthweight is associated with long term effects on health and disease in adult life. Low birthweight is a well established risk factor for adverse long term health, particularly cardiovascular disease and metabolic syndrome [1]. Numerous studies have identified determinants of abnormal birthweight, particularly low birthweight, but also more recently of high birthweight [2]–[4]. Birthweight is used as an indicator of intrauterine growth. The actual pattern of growth in utero can, however, only be estimated by serial ultrasound measurements during pregnancy. Far less is known about determinants of deviating patterns of *growth* in utero than that of abnormal birthweights. Fetal growth is a result of multiple factors including genetic potential for growth, maternal nutrition, maternal metabolism, endocrine factors and placental perfusion and function [5]. In addition, the ability of the fetus to respond to nutrients and other growth regulatory factors may play a role.

The Hyperglycaemia and Adverse Pregnancy Outcomes study (HAPO) has established maternal blood glucose and body mass index (BMI) as independent determinants of large for gestational age (LGA) newborns and excessive body fat at birth [6], [7]. It is implicative in these findings that maternal plasma glucose and other not specified biological factors associated with maternal BMI affect fetal growth and neonatal body composition. The biological mechanisms underlying the effect of glucose on fetal growth are best explained by the Pedersen hypothesis [8]. Pedersen postulated that maternal hyperglycemia was transferred to the fetus, which, in turn, produced and released large amounts of insulin, with fetal hyperinsulinemia as a result. However, the independent effect of BMI is not well explained by any hypotheses that consistently fit observations to the extent that the Pedersen hypothesis does.

Placental function is another potential determinant of fetal growth besides glucose and other BMI-related factors. Placental function includes both transport capacity as well as endocrine and metabolic properties. In principle maternal factors may affect fetal growth via two main pathways. One may operate independently of placenta, i.e. maternal nutrients and other factors enter the fetal circulation directly without any interference from placental tissues. The second pathway affects fetal growth indirectly by modifying placental nutritional transport and metabolism. These two principles are not mutually exclusive. A large number of studies have investigated factors that may affect specific transport mechanisms in placenta, like transfer of glucose, amino acids and fatty acids [9]–[11]. These studies provide strong evidence that placental function (i.e. ability to provide and regulate nutrient supply to the fetus) is modified by both maternal and fetal factors. However, in most clinical and epidemiological investigations like the HAPO–study, the role of placenta has not been specifically addressed. One problem of including placenta is that there is no specific marker that reflects the overall placental function. However, the capacity for nutrient transfer is reflected in the surface area for transport and hence the placental size [12]. Placental weight is a crude marker of placental size, but correlates closely to birthweight in normal pregnancies [13]. Placental weight is widely used as a parameter of placental functional capacity.

The aims of this study were to.

1. Estimate and compare the effects of maternal characteristics on both birthweight and fetal growth in third trimester.
2. Introduce placental weight as a possible determinant of both birthweight and fetal growth in third trimester.

## Methods

### Ethics Statement

Written informed consent was obtained from all participants in the study. All clinical investigations were conducted according to the principles expressed in the Declaration of Helsinki. The study was approved by the Regional Committee for Medical Research Ethics, Southern Norway, Oslo, Norway (S-01191).

### Population

**Figure 1:**
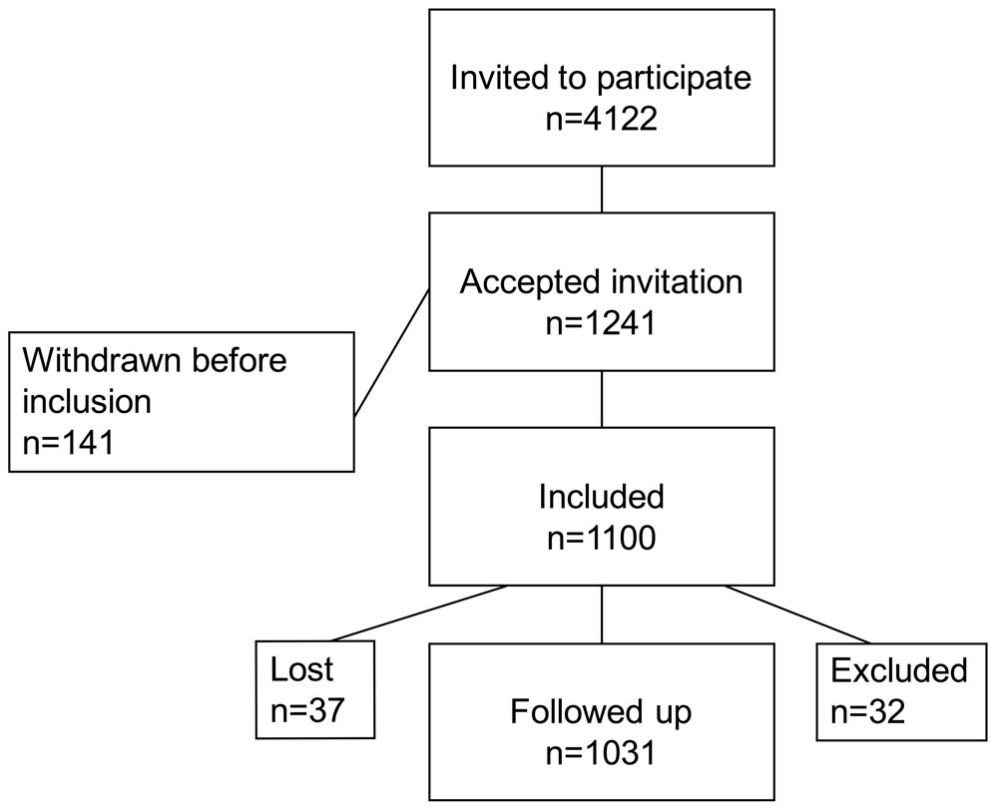
Flow-chart showing the inclusion and exclusion of women in the study.

The STORK study is a prospective cohort study performed in the period 2001–2008. A total of 1031 healthy pregnant women who gave birth at Oslo University Hospital, Rikshospitalet, Norway were included in the study (Figure 1). Study design and data from the first 553 women included in the study have been published [2]–[4]. Inclusion criteria were healthy women of Scandinavian heritage with singleton pregnancies. Exclusion criteria were multiple pregnancies, known pre-gestational diabetes and any severe chronic diseases (lung, cardiac, gastrointestinal or renal). Each pregnant woman had four antenatal visits (visit 1–4), scheduled at weeks 14–16, 22–24, 30–32 and 36–38 of pregnancy. Clinical data and fasting blood samples were collected at each visit. Ultrasound examinations were done at all visits, except the first.

### Independent Variables

Five variables known to influence birthweight were selected based on previous literature and published data on birthweight from the STORK study [2]. The selected variables were maternal age, parity, BMI, gestational weight gain and fasting plasma glucose. In addition placental weight was included as a potential determinant of birthweight and fetal growth.

The same variables were used in all models for comparison.

BMI (kg/m^2^) was calculated by height and weight. Maternal height was measured at the first visit and weight was measured by a calibrated scale at each visit. Gestational weight gain was calculated as the difference between weights measured at visit 4 and visit 1. Measured weight at the first visit was used instead of pre pregnancy weight to avoid false self reporting of pre pregnancy weight. Fasting plasma glucose was measured at weeks 30–32, by Accucheck (Roche Diagnostics, Mannheim, Germany). Due to an unexpected increasing trend in fasting plasma glucose of 0.6 mmol/l over time, the glucose values were adjusted statistically [14]. It was essential to explore to which extent the trend was of biological or analytical origin. The lack of a corresponding increase in the women's body mass index, insulin or age, made it unlikely that the observed trend in blood glucose values could have a biological cause, or be due to selection bias. Thus the time trend in fasting glucose most likely had an analytical cause. Briefly, linear regression gave estimates for the average increase per time unit, under the assumption of a linear increase during the entire period. The glucose values were de-trended according to the coefficients from the linear regression and de-trending of the data removed the increasing trend.

Data on age, parity, obstetric history, educational level and smoking status were registered. Parity was coded as P0 for primigravida and P1 for one or more previous births. Gestational age was based on ultrasound biometric measures made at weeks 17–19. Placental weight including umbilical cord and membranes was measured by the midwife attending the delivery within an hour after delivery.

### Dependent Variables

Both birthweight and estimated fetal growth in third trimester were used as dependent variables. Birthweight was measured by a calibrated scale. Birthweight is given as birthweight for gestational age and sex-specific z-scores [15]. Fetal growth in third trimester was estimated by serial ultrasound measurements. Ultrasound examination was done three times, scheduled at weeks 22–24, weeks 30–32 and weeks 36–38. Each ultrasound examination included fetal biometric parameters. Head circumference (HC), abdominal circumference (AC) and femur length (FL) were measured three times at each visit and the mean value was calculated. The ultrasound examinations were done using an Acuson Aspen ultrasound machine. Estimated fetal weight (EFW) was calculated by Combs formula [16]. Estimated fetal weight in accurate percentiles were calculated according to Norwegian charts developed in Bergen, Norway [17]. Fetal growth in third trimester was chosen as this period is one of rapid fetal growth and fat accumulation, and also the period when metabolic, modifiable factors are most likely to influence fetal growth and fat accumulation [18].

Fetal growth between visit 3 and visit 4 in third trimester was calculated in two ways;

1. The difference in estimated fetal weight percentiles (Δp) according to Norwegian reference charts [17], calculated by Combs formula.
2. The difference in abdominal circumference (ACΔz), head circumference (HCΔz) or femur length (FLΔz) separately. The differences were calculated as differences in z-scores, according to Norwegian reference charts [19].

### Statistical Analysis

Descriptive statistics are presented as mean and standard deviation (SD) and frequency and percentage (%). Maternal determinants for birthweight were explored by univariate and multiple linear regression models. Two models were fitted, one which included only maternal characteristics and one which included the same covariates but adding placental weight.

Variables with p-values <0.1 in the univariate analyses were considered in the multiple models. The same approach was used to explore the associations between maternal characteristics and fetal growth in third trimester. A p-value <0.05 was considered statistically significant. All analyses were done using the Statistical Package for Social Sciences (SPSS version 18.0) for Windows (SPSS Inc., Chicago, IL).

## Results

### Study Population

Demographic and clinical characteristics of the cohort are shown in Table 1. The cohort consisted of 1031 women and their newborns. Mean maternal age was 31.3 years (SD 3.9), 52.9% were primiparous and only 2.7% of the participants were daily smokers during pregnancy. Mean birthweight was 3588 grams (SD 574) and mean gestational age at delivery was 39.5 weeks (SD 1.8, range 26–43 weeks). The study population was comparable to women who gave birth in Oslo on parameters like birthweight, parity, maternal age and gestational age at birth [20].

**Table 1:** Clinical and demographic characteristics of the mothers and babies.

	n	Mean (SD)	%	Range
**Mothers**	1031			
Age (years)		31.3 (3.9)		19–42
Para 0	545		52.9	
BMI visit 1		24.5 (3.9)		17.2–43.9
Weight gain (kg) visit 4–1		10.6 (3.5)		−1.2–29.4
Married or partnership	1011		98.1	
Higher education (≥15 years)	885		85.8	
Daily smoking	28		2.7	
Fasting plasma glucose visit3 (mmol/l)		4.1 (0.45)		2.9–6.2
Gestational diabetes *	56		5.5	
Preeclampsia **	39		3.8	
**Babies**				
Sex (boys)	548		53.1	
Gestational age at birth (weeks)		39.5 (1.8)		26–43
Birthweight (g)		3588 (574)		600–5420
Placental weight (g)		711 (156)		220–1490

* According to WHO-criteria: Plasma glucose ≥7.8 mmol/l 2 hours after an Oral glucose tolerance test of 75 g of glucose.

** Blood pressure ≥140/90 mmHg combined with proteinuria (urinary totalprotein/creatine ratio >30 or +1 on urine dipstick).

### Birthweight

We first examined the association between selected and well known maternal determinants and birthweight. Table 2 shows the variables that were associated with birthweight for gestational age Z-score. The first model did not include placental weight as a covariate.

**Table 2:** Determinants for birthweight-for-gestational age Z-scores*. Results from univariate and multiple linear regression.

				Model 1 (n = 892)			Model 2 (n = 883)		
	Unadjusted B	95% CI	p-value	Adjusted B	95% CI	p-value	Adjusted B	95% CI	p-value
Maternal age (years)	0.015	−0.001–0.03	0.065	0.00	−0.02–0.02	0.98	0.01	−0.02–0.004	0.17
Parity (P1 vs P0)	0.49	0.37–0.61	<0.001	0.46	0.33–0.59	<0.001	0.36	0.25–0.47	<0.001
BMI visit 1	0.06	0.05–0.08	<0.001	0.048	0.03–0.06	<0.001	0.018	0.004–0.03	0.012
Weight gain (kg) visit 1–4	0.05	0.03–0.07	<0.001	0.06	0.04–0.08	<0.001	0.036	0.02–0.05	<0.001
Fasting plasma glucose visit 3 (mmol/l)	0.51	0.37–0.65	<0.001	0.33	0.18–0.48	<0.001	0.22	0.09–0.34	<0.001
Placental weight (100 g)	0.41	0.38–0.44	<0.001				0.39	0.35–0.42	<0.001

* Z-scores according to Norwegian references (15).

Model 1: adjusted for maternal characteristics (age, parity, BMI, weight gain and fasting plasma glucose).

Model 2: adjusted for maternal characteristics (age, parity, BMI, weight gain, fasting plasma glucose) and placental weight.

In the multiple model parity (B 0.46, 95% CI 0.33–0.59, p<0.001), BMI (B 0.048, 95% CI 0.03–0.06, p<0.001), weight gain (B 0.06, 95% CI 0.04–0.08, p<0.001) and fasting glucose (B 0.33, 95% CI 0.18–0.48, p<0.001) remained statistically significant determinants of birthweight, whereas maternal age was not significant (B 0.00, 95% CI −0.02–0.02, p = 0.98). In the model including placental weight as a covariate, placental weight was statistically significant both in the univariate (B 0.41, 95% CI 0.38–0.44, p<0.001) and multiple models (B 0.39, 95% CI 0.35–0.42, p<0.001). In addition, parity (B 0.36, 95% CI 0.25–0.47, p<0.001), BMI (B 0.018, 95% CI 0.004–0.03, p = 0.012), weight gain (B 0.036, 95% CI 0.02–0.05, p<0.001) and fasting plasma glucose (B 0.22, 95% CI 0.09–0.34, p<0.001) remained significant determinants when adjusted for placental weight.

Introducing placental weight as a covariate reduced the effect estimate of the other variables in the model. The magnitude of reduction as estimated by change in the regression coefficient B of the linear regression was 62% for BMI, 40% for weight gain, 33% for glucose and 22% for parity.

### Fetal Growth

Table 3 shows the biometric parameters based on ultrasound measurements at visit 3 and 4, from which fetal growth was estimated. Each measurement is given both in mm and the corresponding z-score for gestational age. In addition, the percentiles of weight estimates are given. Fetal growth between visit 3 and visit 4 was estimated both as difference in percentiles (Δp) and in z-score for each biometric measurement (HCΔz, ACΔz and FLΔz).

**Table 3:** Biometric ultrasound measurements.

	Head circumference(mm)			Abdominal circumference (mm)			Femur length (mm)			Estimated fetalweight (g)		Estimated fetal weight (percentile)	
Gest. weeks	n	Mean (SD)	z-score*	n	Mean (SD)	z-score*	n	Mean (SD)	z-score*	n	Mean (SD)	n	Mean (SD)
30−32(visit 3)	987	288.0 (12.1)	0.15	998	275.7 (16.9)	0.17	984	59.3 (3.2)	0.44	975	1875.0 (248.2)	968	52.0 (25.0)
36−38(visit 4)	943	321.6 (11.6)	−0.19	952	331.6 (19.9)	0.09	941	70.5 (3.1)	0.64	928	2960.0 (355.2)	918	47.3 (26.6)
Δ visit 3−4	910	33.6 (12.7)	−0.34	930	55.9 (18.1)	−0.08	907	11.2 (3.5)	0.20	890	1085.0 (318.9)	877	−4.7 (23.1)

Ultrasound measurements of fetal head circumference, abdominal circumference and femur length measured at two times in the third trimester. Fetal growth was calculated as the difference between measurements at gestational weeks 30–32 and 36–38, respectively.

N varies due to missing numbers.

Measurements of head circumference, abdominal circumference and femur length are given in mm and reported as means and SD.

* Z-scores calculated according to Norwegian referance charts (19).

The differences between visit 3 and visit 4 are given as differences in mm or grams and as difference in z-scores or percentiles.

Table 4 shows the results of the linear regression analyses when fetal growth in third trimester was estimated as differences in estimated fetal weight percentiles between visit 3 and visit 4. Univariate analyses showed that parity (B 4.35, 95% CI 1.29–7.41, p = 0.005), BMI (B 0.71, 95% CI 0.32–1.11, p<0.001) and weight gain (B 0.62, 95% CI 0.17–1.07, p = 0.007) were statistically significant, whereas maternal age (B 0.11, 95% CI −0.29–0.50, p = 0.6) and fasting glucose (B 2.55, 95% CI 0.89–5.99, p = 0.15) were not significant. In the multiple model parity (B 4.73, 95% CI 0.16–8.07, p = 0.006), BMI (B 0.64, 95% CI 0.23–1.06, p = 0.002) and weight gain (B 0.70, 95% CI 0.24–1.16, p = 0.003) remained significant. When placental weight was included, parity (B 3.9, 95% CI 0.75–7.0, p = 0.015), BMI (B 0.49, 95% CI 0.08–0.89, p = 0.02), weight gain (B 0.60, 95% CI 0.15–1.06, p = 0.01) and placental weight (B 2.08, 95% CI 1.03–3.13, p<0.001) remained significant, also in the multiple model. Introducing placental weight reduced the effect of BMI on fetal growth by 23%, weight gain by 14% and parity by 17%, as estimated by change in B.

**Table 4:** Determinants for fetal growth in third trimester.

				Model 1 (n = 847)			Model 2 (n = 829)		
	Unadjusted B	95% CI	p-value	Adjusted B	95% CI	p-value	Adjusted B	95% CI	p-value
Maternal age (years)	0.11	−0.29–0.50	0.6						
Parity (P1vs P0)	4.35	1.29–7.41	0.005	4.73	0.16–8.07	0.006	3.9	0.75–7.0	0.015
BMI visit 1	0.71	0.32–1.11	<0.001	0.64	0.23–1.06	0.002	0.49	0.08–0.89	0.02
Weight gain (kg)	0.62	0.17–1.07	0.007	0.70	0.24–1.16	0.003	0.60	0.15–1.06	0.01
Fasting glucose(mmol/l)	2.55	−0.89–5.99	0.15						
Placental weight (100g)	2.60	1.60–3.60	<0.001				2.08	1.03–3.13	<0,001

Results from univariate and multiple linear regression using difference in estimated weight percentiles* as fetal growth (Δp4–p3).

* Estimated fetal weight percentiles according to Norwegian reference charts (17).

Model 1: adjusted for maternal characteristics (maternal age, parity, BMI, weight gain and fasting plasma glucose).

Model 2: adjusted for maternal characteristics (maternal age, parity, BMI, weight gain and fasting plasma glucose) and placental weight.

Finally, we analysed the effects of the selected characteristics on fetal growth made on each of the fetal biometric measures.

### Abdominal Circumference

Table 5 shows the effects of determinants on the growth of the abdominal circumference between visit 3 and visit 4. In the univariate analyses all variables except maternal age were significant. In the multiple model parity (B 0.18, 95% CI 0.07–0.29, p = 0.001), BMI (B 0.022, 95% CI 0.005–0.03, p = 0.002) and weight gain (B 0.026, 95% CI 0.008–0.04, p = 0.001) remained statistically significant, whereas fasting glucose (B 0.044, 95% CI −0.08–0.17, p = 0.48) no longer was a statistically significant independent variable for increase in abdominal circumference. Including placental weight to the model showed that placental weight was a significant determinant (B 0.095, 95% CI 0.06–0.13, p<0.001). In the multiple model fasting plasma glucose was not significant (B 0.016, 95% CI −0.11–0.14, p = 0.8). BMI reached borderline significance (B 0.014, 95% CI 0.00–0.028, p = 0,048) as an independent determinant when adjusted for parity, weight gain and placental weight. For growth of the fetal abdominal circumference BMI showed the largest reduction in B (36%) when placental weight was included.

**Table 5:** Determinants for fetal abdominal growth in third trimester.

				Model 1 (n = 872)			Model 2 (n = 852)		
	Unadjusted B	95% CI	p-value	Adjusted B	95% CI	p-value	Adjusted B	95% CI	p-value
Maternal age (years)	0.002	−0.01–0.02	0.77						
Parity P1 vs P0	0.17	0.07–0.27	0.001	0.18	0.07–0.29	0.001	0.14	0.034–0.24	0.009
BMI visit 1	0.023	0.01–0.04	0.001	0.022	0.005–0.03	0.002	0.014	0.00–0.028	0.048
Weight gain (kg)	0.02	0.005–0.04	0.009	0.026	0.008–0.04	0.001	0.021	0.005–0.036	0.008
Fasting glucose (mmol/l)	0.13	0.01–0.24	0.031	0.44	–0.08–0.17	0.48	0.016	–0.11–0.14	0.8
Placental weight (100 g)	0.11	0.08–0.14	<0.001				0.095	0.06–0.13	<0.001

Results from univariate and multiple linear regression using difference in abdominal circumference in z-scores* as abdominal growth (ACΔz).

* Z-scores according to Norwegian reference charts (19).

Model 1: adjusted for maternal characteristics (maternal age, parity, BMI, weight gain and fasting plasma glucose).

Model 2: adjusted for maternal characteristics (maternal age, parity, BMI, weight gain and fasting plasma glucose) and placental weight.

### Head Circumference and Femur Length

In univariate analyses of HCΔz only BMI was a significant determinant. For FLΔz placental weight was the only significant determinant (data not shown).

## Discussion

The current study has two main findings. First, we show that parity, BMI and weight gain, but not fasting glucose, were significant determinants of intrauterine *growth* in third trimester, both when calculated as a composite consisting of fetal head, abdomen and femur length (estimated weight) and when measured as increase in the abdominal circumference.

Secondly, placental weight was identified as a significant independent determinant both for birthweight and for fetal growth in third trimester. The latter finding was valid both for the abdominal circumference and for estimated fetal weight. The present study also confirms that parity, BMI, weight gain and fasting glucose were determinants of birthweight, which is in accordance with several previous studies [2], [6].

There is accumulating evidence that the nutritional state of the pregnant women at the start of pregnancy is important for several measures of pregnancy outcome, including birthweight [21]. BMI at the beginning of pregnancy may be considered as a surrogate for the nutritional status of the mother. BMI has been shown to be a predictor of large for gestational age neonates (LGA) in several studies including the HAPO-study [7]. In the current study BMI was found to be an independent determinant also of fetal *growth* in third trimester. BMI is an index consisting of both height and weight, and is associated with a number of more specific biological variables including genetic, nutritional and endocrine factors that all may affect fetal growth. In particular substances released from the maternal adipose tissue may affect fetal growth either directly or via modification of placental functions. In the current study we did not aim to identify effects of specific factors associated with BMI on fetal growth, except glucose. We have previously, however, reported that interleukin -1 receptor antagonist (IL1-Ra) is a determinant of birthweight [22]. IL1-Ra is one of the factors released from adipose tissue [23].

Weight gain during pregnancy is related to fetal growth. Low weight gain is associated with small for gestational age neonates (SGA) [24], and high weight gain with birthweight above 4200 and 4500 grams as well as LGA [2], [25]. Here we showed that weight gain also was an independent determinant of third trimester *growth*.

Parity was identified as a determinant for fetal growth. It is well described that parity influences birthweight. The magnitude of reduced birthweight is about 200 g for the first born child [26]. We showed that fetal growth in third trimester was influenced by the parity of the mother. The effect of parity might be linked to the capacity of the spiral arteries to fully dilate or be invaded by trophoblasts which differ between first and subsequent pregnancies [27]. The concept that growth pattern differs between the first and a later born child may be illustrated by data suggesting that truncal obesity is more common in the first born child and that firstborn carries a higher metabolic risk in early adulthood [28], [29].

Fasting plasma glucose measured in third trimester was significantly associated with birthweight in our study. Since transport of glucose is dependent on the maternal-fetal gradient across the placenta, we expected fasting glucose in third trimester to play an important role for fetal *growth* measured during the period of rapid fetal growth in third trimester. We found, however, that maternal fasting glucose in week 30–32 failed to reach statistical significance. This finding may apparently be inconsistent with several previous studies of determinants of birthweight categories including the HAPO-study. However, the current and previous findings are not necessarily contradictory. The lack of effect of glucose on fetal growth could be explained by considering the body composition of the baby. We measured fetal growth as either change in a composite variable consisting of fetal head, abdomen and femur or just change in the abdominal circumference. Fasting glucose had no effect on neither of these endpoints. We therefore speculate that the effect of hyperglycaemia preferentially leads to deposition of fat in the extremities and the thoracic truncus of the fetus and hence will not be captured by either measuring fetal growth as abdominal circumference or estimated fetal weight as calculated in the present study, but will be reflected in the birthweight. This notion is supported by preliminary data showing that caliper based skin fold measures of arms, legs and the sub scapular area were significantly correlated with fasting plasma glucose. In fact, the association remained significant after adjusting for the other maternal determinants used in our study (data not shown).

Placental weight has been shown to be closely correlated with birthweight in large studies [13]. In our study placental weight and birthweight were also strongly correlated (r = 0.67, p<0.001) and we also showed that placental weight was independently associated with fetal *growth* in third trimester. This finding is not surprising, placenta being responsible for all maternal-fetal oxygen and nutrient exchange. However, in the large majority of previous studies of determinants of birthweight, including the HAPO-study, placental weight has not been considered. Placenta exhibits a linear growth throughout pregnancy [30], [31].Thus, given that placental weight reflects functional properties, final placental weight should also provide information about placental function earlier in pregnancy. Placental growth includes both lateral growth of the chorionic disc and thickness of placenta. Lateral growth reflects the uterine area covered by the placenta and hence how many spiral arteries are potential suppliers of the placenta. The thickness of the placenta reflects the arborization of the villous tree and vascular nutrient exchange surface [32].

Placental volume can be estimated by ultrasound. In a study by Thame et al placental volume was estimated by ultrasound at week 20 and found to be positively correlated with placental weight at birth (r = 0.46, p<0.001) [33]. Maternal characteristics have been shown to influence growth of placenta and also various dimensions of placental growth. In a study of more than 24000 placentas in the US the relationships between maternal characteristics, placental growth measures and birthweight were explored. Placental weight alone accounted for 36.5% of birthweight variation, whereas only 13.9% of birthweight variation could be explained by maternal characteristics like age, parity, height and weight, cigarette use, ethnicity and socio-economic status [13]. Our data gave similar results; the selected variables parity, BMI, weight gain and fasting glucose explained 16% of variation in birthweight, whereas placental weight alone explained 39% of variation in birthweight (data not shown).

In a study of the US cohort referred to above, maternal characteristics and associations to three dimensions of placental growth (placental weight, thickness and chorionic plate area) were studied [34]. Pre pregnancy BMI and weight gain were identified as predictors of hypertrophy of all three dimensions of placental growth. Thus, currently available data indicate that effects of BMI and weight gain on birthweight and fetal growth at least partly is mediated through an effect on placental properties, including growth. This notion is supported by the fact that BMI had an independent effect on placental weight (p<0.001, data not shown), but also on birthweight with placental weight as a covariate. A better estimation of the magnitude of direct and indirect effects may be obtained by a path analysis [22]. There are several possible biological reasons for the reduced effects of the maternal characteristics on birthweight when placental weight is included in the models. The selected maternal characteristics may exert their effects on growth by affecting transplacental transport. Transplacental transport is affected by blood flow both on maternal and fetal side. Furthermore, the number and efficiency of placental transport proteins may also be influenced by maternal factors. In addition, it can not be excluded that placental production of growth factors may be affected.

### Strengths and Limitations

The strengths of this study are the well characterised cohort in terms of maternal characteristics and the longitudinal design including serial ultrasound measurements. This allowed us to explore the associations between several maternal characteristics and intrauterine fetal growth as the endpoint, in addition to the more conventional way of measuring fetal growth as birthweight. We did not obtain weight of the placenta after being trimmed for membranes and umbilical cord. There is however a close correlation (r = 0.98) between the weight of trimmed and untrimmed placentas [35].

It can be argued whether conditional growth percentiles should be used. In separate analyses we calculated conditional growth between visit 3 and visit 4 and obtained conditional percentiles which were used in the linear regression model (results not shown). Although the regression coefficients were different, the variables that remained statistically significant in the multiple model were exactly the same as using unconditional growth percentiles. We acknowledge that using conditional growth gives an interesting and probably more correct understanding of fetal growth. However, we have experienced that the concept of conditional growth represents a challenge to understand. We therefore chose to use the unconditional percentiles as the results are the same in the current work.

### Conclusion

Maternal metabolic factors including BMI, weight gain and glucose values are modifiable determinants of fetal growth. Avoiding abnormal fetal growth may reduce the number of newborns experiencing adverse outcome both in short and long term. However, our findings indicate that placental weight markedly modifies the effect of determinants on both birthweight and fetal growth parameters. The differential effect of third trimester glucose on birthweight and growth parameters illustrates that birthweight and fetal growth are not identical entities in terms of their determinants.
